# Comparing immobilisation devices in gynaecological external beam radiotherapy: improving inter‐fraction reproducibility of pelvic tilt

**DOI:** 10.1002/jmrs.804

**Published:** 2024-06-19

**Authors:** Shimon Prasad, Linda J. Bell, Benjamin Zwan, Florence Ko, Tayla Blackwell, Kevin Connell, Cameron Stanton, Meegan Shepherd, John Atyeo, Mark Stevens, Marita Morgia

**Affiliations:** ^1^ Northern Sydney Cancer Centre Radiation Oncology Department, Royal North Shore Hospital St Leonards New South Wales Australia; ^2^ School of Mathematical and Physical Sciences University of Newcastle Newcastle New South Wales Australia; ^3^ Faculty of Medicine and Health The University of Sydney Sydney New South Wales Australia

**Keywords:** Gynaecological cancer, pelvic tilt, radiotherapy, stabilisation devices

## Abstract

**Introduction:**

The aim was to determine which immobilisation device improved inter‐fraction reproducibly of pelvic tilt and required the least pre‐treatment setup and planning interventions.

**Methods:**

Sixteen patients were retrospectively reviewed, eight immobilised using the BodyFIX system (BodyFIX®, Elekta, Stockholm, Sweden) and eight using the Butterfly Board (BB) (Bionix Radiation Therapy, Toledo, OH, USA). The daily pre‐treatment images were reviewed to assess setup variations between each patient and groups for pelvic tilt, pubic symphysis, sacral promontory and the fifth lumbar spine (L5).

**Results:**

Compared with the planning CT, pelvic tilt for most patients was within ±2° using the BodyFIX and ± 4° for the BB. The Butterfly Board had a slightly higher variance both for patient‐to‐patient (standard deviation of the systematic error) and day‐to‐day error (standard deviation of the random error). Variance in position between individual patients and the two stabilisation devices were minimal in the anterior–posterior (AP) and superior–inferior (SI) direction for the pubic symphysis, sacral promontory and L5 spine. Re‐imaged fractions due to pelvic tilt reduced by about half when BodyFIX was used (39.1% BB, 19.4% BodyFIX). One patient treated with the BB required a re‐scan for pelvic tilt. Three patients required a re‐scan for body contour variations (two using BodyFIX and one with the BB).

**Conclusions:**

BodyFIX resulted in a more accurate inter‐fraction setup and efficient treatment and is used as the standard stabilisation for gynaecological patients at our centre. It reduced the pelvic tilt variance and reduced the need for re‐imaging pre‐treatment by half.

## Graphical abstract

The use of the BodyFIX system reduced the pelvic tilt variance for gynaecological patients. This form of immobilisation also reduced the need for re‐imaging pre‐treatment by half, reducing imaging dose to the patients. Approximately 82% of departments who responded to the study survey reported seeing pelvic tilt on pre‐treatment imaging indicating that this is an issue worth finding a strategy to combat. The BodyFIX system could result in more accurate and efficient treatment of gynaecological external beam radiotherapy patients.

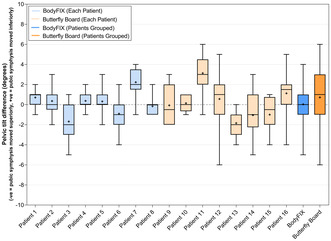

## Introduction

Radical external beam radiotherapy (EBRT) for locally advanced cervical cancer (LACC) targets both the primary site of cancer and regional lymph nodes (RLN), and for high‐risk endometrial cancer (hrEC) the post‐operative central pelvis and RLN are targeted.^1^ Either combined with synchronous chemotherapy, or as monotherapy, high‐dose EBRT in both these scenarios can result in clinically significant gastrointestinal (GI) and genitourinary (GU) toxicity.^2^, ^3^ Anatomic proximity of target structures (LACC: intact uterus, cervix, upper vagina and parametria; hrEC: residual parametria and vaginal vault) and pelvic organs at risk (OAR) within complex‐shaped and elongated RLN fields are major determinants of acute and emergent (late) treatment toxicity.^4^

The use of highly conformal EBRT techniques such as intensity‐modulated radiotherapy (IMRT) and volumetric modulated arc therapy (VMAT) has enabled higher dose conformity to the target structures while decreasing the dose to critical structures like the bladder, rectum and small bowel.^5^, ^6^ While highly conformal techniques deliver increased target coverage and potentially decrease toxicity, both internal organ motion and setup variability can have dosimetric and clinical impact on the treatment delivered to patients. Changes in the bladder and rectal volumes, rectal gas and tumour changes have all been identified as potential causes of inter‐fraction motion in gynaecological patients.^7^, ^8^ Treatment volumes are long to encompass both the target organ and the lymph nodes which makes patient stabilisation and bowel and bladder preparation important to ensure correct alignment for radiotherapy treatment.

Two different stabilisation setups are available to stabilise pelvic radiotherapy patients at our department. The first includes a Butterfly Board (Bionix Radiation Therapy, Toledo, OH, USA) that is used to stabilise the arms above their head, and a SofTouchTM knee wedge under the knees and foot block (QFix, USA) for the feet to rest in. The second system uses the BodyFIX system (BodyFIX®, Elekta, Stockholm, Sweden), which is a full‐body vacbag without a drape. The patient lies on top of the vacbag with arms positioned above the head. The vacbag is moulded under and around the sides of the patient to support them from head to toes with the pelvic area supported.

The aims of this retrospective study were to: (i) determine which immobilisation device improved inter‐fraction reproducibly of pelvic tilt during pre‐treatment setup prior to radiotherapy treatment, (ii) determine which immobilisation device required the least pre‐treatment and planning interventions, and (iii) survey which stabilisation devices other departments use for gynaecological radiotherapy and their experience with pelvic tilt.

## Methods

An ethics exemption was granted for this quality assurance/quality improvement project by the Northern Sydney Local Health District Human Research Ethics Committee.

A total of 16 patients were retrospectively selected for the study and had been prescribed an external beam dose of 45‐55Gy in 25 fractions. Sixteen consecutively treated patients were selected, eight patients had been setup using the BodyFIX system (BodyFIX®, Elekta, Stockholm, Sweden), which is a full‐body vacbag, and eight were stabilised using a Butterfly Board (Bionix Radiation Therapy, Toledo, OH, USA), SofTouch™ knee wedge and foot block (QFix, USA). Planning CT scans were acquired helically with a 2‐mm slice thickness and 600‐mm field of view (512 × 512 pixel matrix) at 120 kVp on a Brilliance Big Bore scanner (Philips Medical Systems, Cleveland, OH, USA). The scan extended from the tenth thoracic spine to 15 cm below the ischial tuberosity. Additional staging imaging studies, for example fluorodeoxyglucose‐positron emission tomography (FDG‐PET)/pelvic magnetic resonance imaging (MRI) for patients with LACC, and vaginal iodated contrast in all patients at the time of planning CT, aided the definition of clinical target volumes (CTV) and OARs. Bladder preparation was determined by Radiation Oncologist specific protocols. Bladder preparation for planning CT and treatment was evenly distributed between bladder full and empty.

### Setup variance assessment

Daily image guidance using a combination of kilovoltage (kV) kV/kV paired, cone beam computed tomography (CBCT) and dual images consisting of a CBCT and a kV/kV paired imaging was conducted as per department protocol. CBCT would be taken on the first three fractions then once weekly with all other fractions having kV/kV paired imaging acquired. Dual imaging was taken on patients where the treatment volumes were longer than the field of view available on the CBCT. The first pre‐treatment images for each treatment fraction were retrospectively reviewed in Offline Review (Varian Medical Systems, Palo Alto, CA, USA) to assess setup variations between anatomical points. Six degrees of freedom matching was not available at the time within our department either on the machine or in Offline Review, so only four degrees of freedom correction were available. Subsequent images taken due to setup variance on the initial image were not included in the setup variance assessment. Setup variance requiring re‐setup and re‐imaging included any variance which caused the soft tissue inside the clinical target volume to move outside of the planning target volume and body contour changes larger than 2 cm. For fractions where dual imaging was acquired, the CBCT image was used primarily for assessment.

Measurements of pelvic tilt, pubic symphysis, sacral promontory and the fifth lumbar spine (L5) were taken on the planning reference image and the daily acquired pre‐treatment image by one of four radiation therapists. All measurements were made using the measurement tool available in Offline Review (Fig. S1A). Pelvic tilt was calculated by measuring the angle between the sacral promontory and the superior‐anterior corner of the pubic symphysis. Both magnitude and direction were measured with positive measurements indicating the pubic symphysis had moved superiorly and a negative value indicating an inferior movement. Measurements of the pubic symphysis variations between the planning reference image and the daily acquired image were measured from the superior‐anterior corner of the pubic symphysis in both the anterior–posterior (AP) and superior–inferior (SI) directions. The variation in the sacral promontory was also measured in the AP and SI directions. If the superior‐anterior corner of the L5 spine was visible on both the planning reference image and the daily acquired image, the AP and SI variation was also measured. Positive values for the pubic symphysis, sacral promontory and L5 spine indicate that the structure was either moving anteriorly or superiorly and negative values indicated a posterior or inferior movement.

To determine the difference between these measurements for the two stabilisation devices, descriptive statistics were used. Firstly, the difference between the measurements for each patient and for each stabilisation group were assessed using box and whisker plots. In addition to this, systematic and random errors were calculated per patient, defined as the patient's average variation and standard deviation of the variations across all fractions respectively.^9^ The overall mean or group systematic error (M) was calculated. The standard deviation of the systematic error (∑) was used to calculate patient‐to‐patient variation (inter‐patient), and the standard deviation of the random error (σ) was used to calculate the day‐to‐day variation (inter‐fraction).

### Pre‐treatment re‐imaging, planning image reviews and re‐scanning

For fractions where re‐setup was required, the re‐imaging prior to treatment was reviewed. The treatment records for all patients were reviewed to determine whether an image review by planning or a simulation re‐scan was requested during treatment. The number of re‐imaging, planning image reviews and simulation re‐scanning instances were calculated, and the reasons for these requests were determined on review. The variation in these parameters was compared between the two stabilisation device cohorts.

### Survey

The link to a Survey Monkey survey (Table S1A) consisting of seven questions was emailed to all radiation therapy educators within the public hospitals in New South Wales and the Australian Capital Territory. This survey was developed by the gynaecological tumour stream and sought information about the way departments setup their gynaecological patients for external beam radiotherapy, their experience with pelvic tilt and perceived causes for pelvic tilt. The survey consisted of mainly multiple‐choice questions with space in five of the questions for free text to gain more detailed information. The survey was reviewed by all members of the gynaecological tumour stream and an independent radiation therapist before the survey was distributed.

## Results

### Patient demographics

Patient demographics are displayed in Table 1. The patient age ranged from 28 to 90 years with a median age of 61 years. Twelve patients were diagnosed with LACC, three with hrEC and one with cancer of the fallopian tube. Similar numbers of LACC and hrEC were in each group of patients. There was a range of International Federation of Gynaecology and Obstetrics (FIGO) stages ranging from IB to IIIC1. The same number of patients undergoing definitive and post‐operative radiotherapy were observed in each group and more patients were undergoing concurrent chemotherapy in the butterfly board group.

**Table 1:** Patient demographics.

		All patients	BodyFix patients	Butterfly Board patients
Cancer type	LACC	12	6	6
	hrEC	3	1	2
	Fallopian tube	1	1	0
FIGO staging	IB	1	0	1
	IB1/2	1	1	0
	II	2	1	1
	IIB	6	2	4
	IIIA	1	0	0
	IIIB	3	2	1
	IIIC	1	0	1
	IIIC1	1	1	0
Cancer site	Cervix uteri	10	4	6
	Endocervix	2	2	0
	Uterine adnexa	1	1	0
	Endometrium	2	0	2
	Fallopian tube	1	1	0
Definitive or post‐operative	Definitive	12	6	6
	Post‐operative	4	2	2
Concurrent chemotherapy	Yes	11	4	7
	No	5	4	1
Age (years)	Median	61	65	55
	Range	28–90	37–90	28–74
Bladder preparation	Bladder empty	8	4	4
	Bladder full	7	3	4
	Clamped catheter	1	1	0

FIGO, International Federation of Gynaecology and Obstetrics; hrEC, high‐risk endometrial cancer; LACC, locally advanced cervical cancer.

### Image assessment

A total of 400 pre‐treatment images, one for each fraction delivered, were available for review consisting of 191 kV/kV pairs, 141 CBCT scans and 68 dual images consisting of both CBCT and kV/kV pairs. The majority (56%) of the patients had a CTV volume that extended to L4 or above.

### Setup variance assessment

#### Pelvic tilt

**Figure 1:**
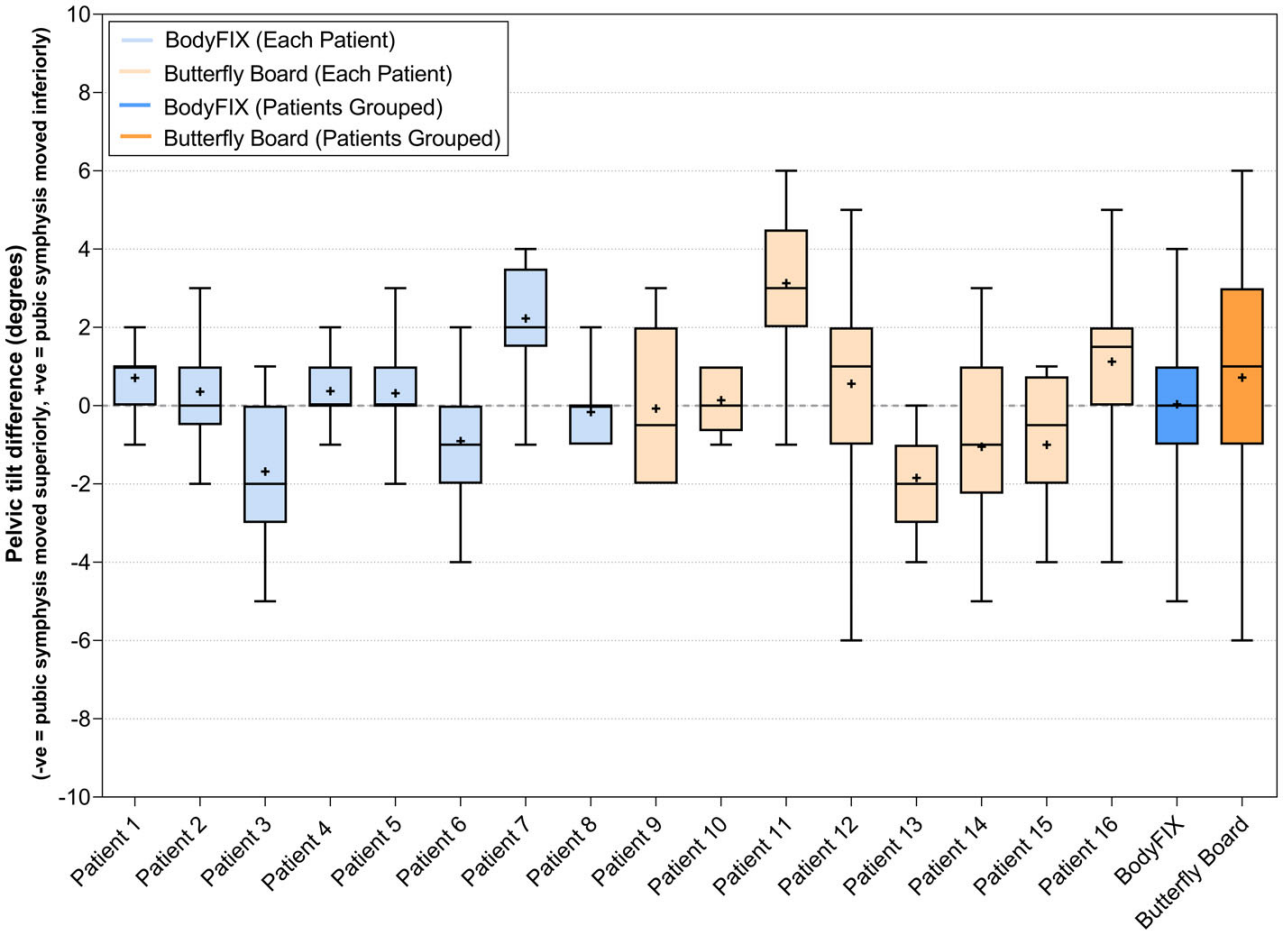
Pelvic tilt variation between the planning scan and pre‐treatment imaging for the BodyFIX and Butterfly Board setups for each patient and as a group. Whiskers indicate the range; the line is the median and the + is the mean.

**Figure 2:**
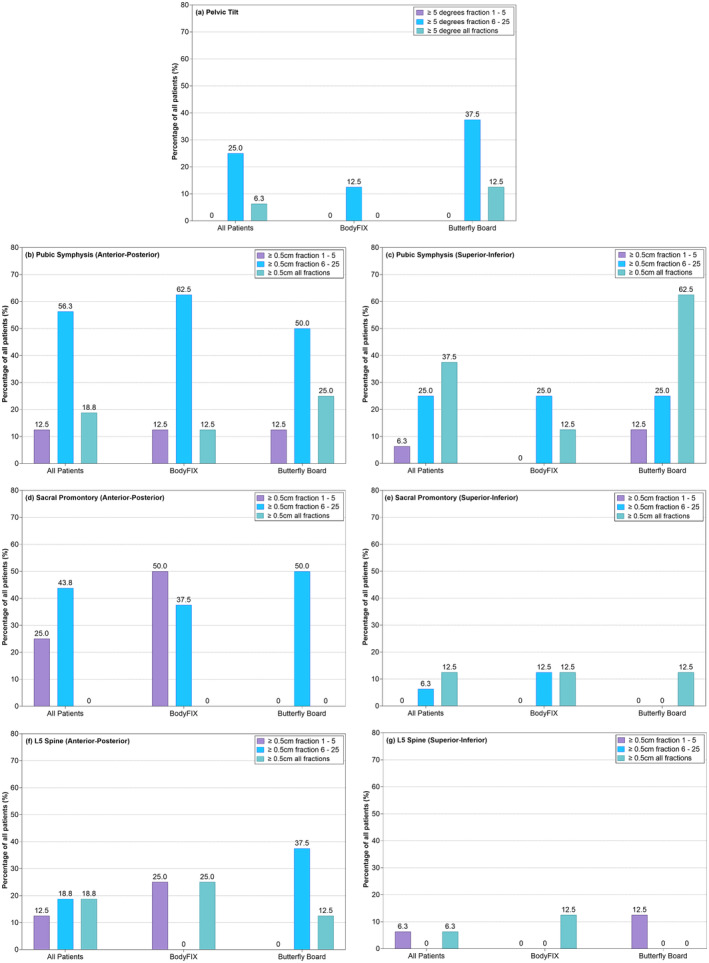
The percentage of patients that display a ≥5° or 0.5 cm absolute difference between the planning CT and the pre‐treatment imaging for both BodyFIX and Butterfly Board setups for the pelvic tilt (a), pubic symphysis (b, e), sacral promontory (c, f), L5 spine (d, g) during fraction 1–5 (purple), fraction 6–25 (blue) and during all fractions (green).

The pelvic tilt variation is displayed in Figure 1 for each patient and each stabilisation device. The majority of patients were within the range of ±2° when using the BodyFIX setup (88%) compared with ±4° for the Butterfly Board setup (90%). The Butterfly Board also had a slightly higher variance both for patient‐to‐patient (standard deviation of the systematic error) and day‐to‐day error (standard deviation of the random error) (Table 2). An absolute difference of ≥5° was detected in only fraction 6–25 for the BodyFIX system and predominately in fraction 6–25 for the Butterfly Board (Fig. 2a).

**Table 2:** Setup error for each measurement point for each stabilisation devices.

Measurement point	Stabilisation device	Median (range)*	Overall systematic error, M	SD of systematic error, ∑	SD of random error, σ
Pelvic tilt	BodyFIX	0.0° (−5.0 to 4.0°)	0.16°	1.15°	1.21°
	Butterfly Board	0.0° (−6.0 to 6.0°)	0.12°	1.55°	1.85°
Pubic symphysis (AP)	BodyFIX	0.1 cm (−0.9 to 2.2 cm)	0.05 cm	0.15 cm	0.32 cm
	Butterfly Board	0.0 cm (−1.1 to 2.0 cm)	−0.06 cm	0.16 cm	0.29 cm
Pubic symphysis (SI)	BodyFIX	0 cm (−0.8 to 1.3 cm)	0 cm	0.18 cm	0.22 cm
	Butterfly Board	−0.1 cm (−0.8 to 0.8 cm)	−0.06 cm	0.15 cm	0.27 cm
Sacral promontory (AP)	BodyFIX	0 cm (−0.8 to 1.2 cm)	−0.02 cm	0.11 cm	0.25 cm
	Butterfly Board	0 cm (−0.9 to 0.5 cm)	−0.03 cm	0.15 cm	0.20 cm
Sacral promontory (SI)	BodyFIX	−0.1 cm (−0.7 to 0.8 cm)	−0.05 cm	0.06 cm	0.19 cm
	Butterfly Board	−0.1 cm (−0.9 to 0.5 cm)	−0.08 cm	0.06 cm	0.16 cm
L5 Spine (AP)	BodyFIX	−0.1 cm (−0.7 to 1.0 cm)	−0.05 cm	0.16 cm	0.18 cm
	Butterfly Board	0.1 cm (−0.5 to 0.6 cm)	0.06 cm	0.14 cm	0.18 cm
L5 spine (SI)	BodyFIX	0.0 cm (−0.8 to 0.6 cm)	−0.05 cm	0.08 cm	0.14 cm
	Butterfly Board	−0.1 cm (−0.5 to 0.2 cm)	−0.09 cm	0.05 cm	0.12 cm

AP, anterior–posterior; cm, centimetres; L5, Fifth lumbar vertebrae; SI, superior–inferior.

* Does not account for repeated measures.

#### Pubic symphysis

**Figure 3:**
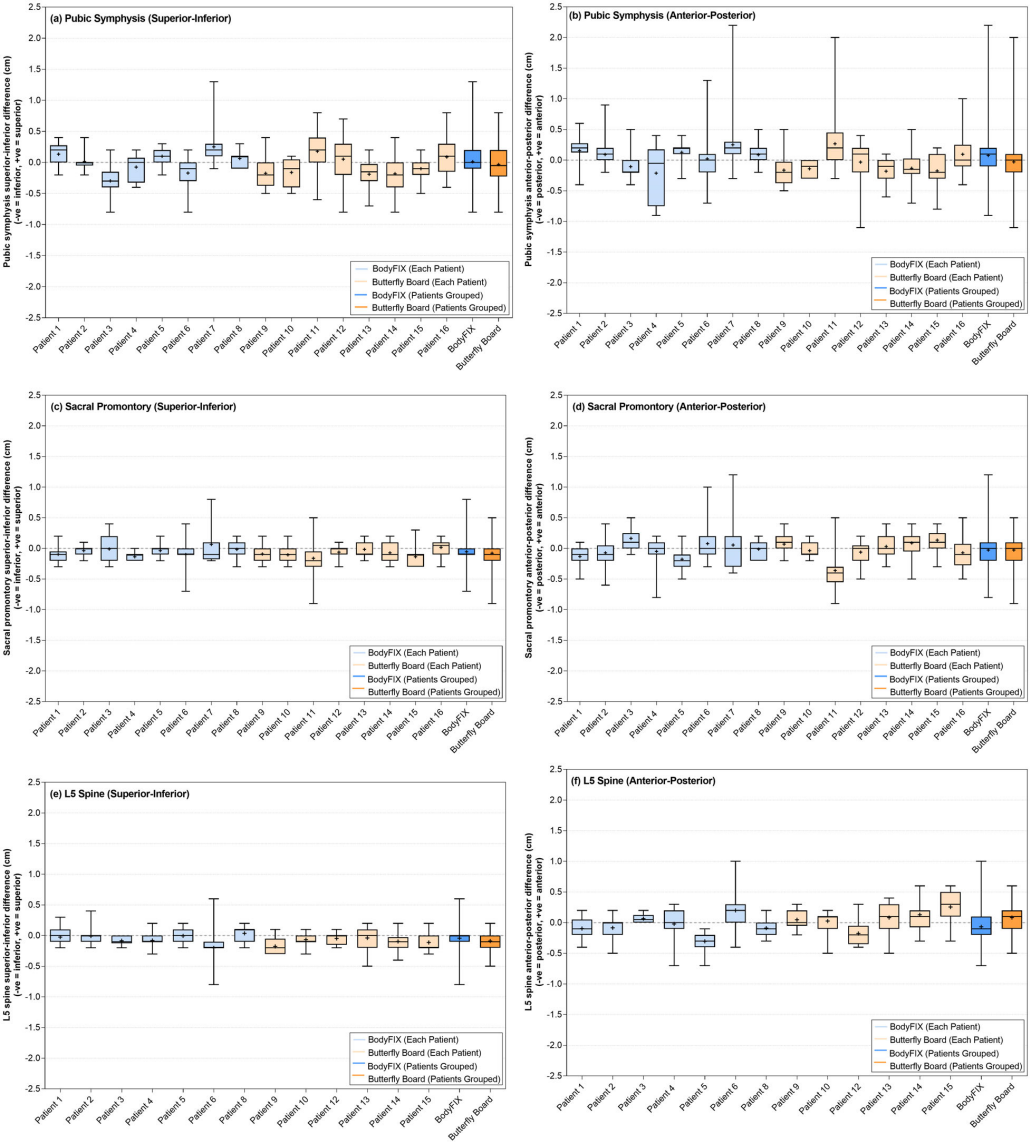
Variation in the location of the pubic symphysis in the superior–inferior direction (a) and anterior–posterior direction (b), sacral promontory in the superior–inferior (c) and anterior–posterior direction (d), and L5 spine in the superior–inferior direction (e), and anterior–posterior direction (f) on the planning CT and the pre‐treatment imaging for the BodyFIX (blue) and Butterfly Board (orange) setups for each patient and as a group. Whiskers indicate the range; the line is the median and the + is the mean.

The variation of the alignment of the pubic symphysis is illustrated in the SI (Fig. 3a) and AP (Fig. 3b) directions between each patient and the two stabilisation techniques. There was little difference in the variance in the stabilisation devices from patient to patient and day to day (Table 2). An absolute difference of ≥0.5 cm in the AP direction was detected predominately in fraction 6–25 for both the BodyFIX and Butterfly Board (Fig. 2b). There was also a higher rate of ≥0.5 cm absolute difference in both the 1–5 fraction and 6–25 fraction time period for the Butterfly Board group. When assessing the absolute difference in the SI direction, there was more differences ≥0.5 cm in both the 1–5 fraction and 6–25 fraction patient group stabilised with the Butterfly Board (Fig. 2c).

#### Sacral promontory

The difference between the alignment of the sacral promontory in the SI and AP directions for each patient and either stabilisation device is displayed in Figure 3c and d. Very little difference was seen in the patient‐to‐patient and day‐to‐day variance between the two devices (Table 2). When assessing absolute differences of ≥0.5 cm in the sacral promontory position in the AP direction, the patients stabilised with the BodyFIX system showed a larger rate of difference in the 1–5 fraction time period only, whereas the Butterfly Board patients had more difference in the 6–25 fraction period only (Fig. 2d). In the SI direction absolute difference was displayed in both fraction 1–5 and fraction 6–25 time periods for the Butterfly Board only, whereas the BodyFIX system resulted in an equal number of patients having differences in only the fraction 6–25 time period to patients having difference in both the fraction 1–5 and fraction 6–25 time period (Fig. 2e). The sacral promontory also had less movement ≥0.5 cm in the SI direction when compared to the AP direction (Fig. 2d and e).

#### L5 spine

The L5 spine was visualised on seven patients (147 images) stabilised with the BodyFIX system and 6 patients (125 images) with the Butterfly Board. The variance in the setup for each patients and the stabilisation devices for the L5 spine are displayed in Figure 3 for the SI (Fig. 3e) and AP (Fig. 3f) directions. There was also little difference in the variance in the stabilisation devices from patient to patient and day to day (Table 2). Absolute differences of ≥0.5 cm were detected in the AP direction in the 1–5 fraction period in 25% of patients treated using the BodyFIX system and a further 25% of patients had differences in the fraction 1–5 and fraction 6–25 time period. The Butterfly Board measured a larger rate of absolute difference in fraction 6–25 only (Fig. 2f). In the SI direction, if a patient displayed an absolute difference of ≥0.5 cm, they were more likely to have this difference in the fraction 1–5 and 6–25 time periods if stabilised with the BodyFIX system, whereas these differences were more likely to only occur in the 1–5 fraction period for those stabilised using the Butterfly Board (Fig. 2g). Less movement ≥0.5 cm in the L5 spine was also reported in the SI directions when compared to the AP direction (Fig. 2f and g).

### Pre‐treatment re‐imaging, planning image reviews and re‐scanning

#### Pre‐treatment re‐imaging

Re‐imaging before treatment was required for 13 patients in total and for 13.5% of all fractions, with 15.5% of fractions using the BodyFIX system and 11.5% using the Butterfly Board requiring re‐imaging (Table 3). 27.8% of instances of re‐imaging were due to pelvic tilt. 14.8% of instances of re‐imaging were due to FSD variations. Re‐imaged fractions due to pelvic tilt reduced by about half when BodyFIX was used (39.1% for Butterfly Board compared with 19.4% for BodyFIX). However, re‐imaged fractions due to FSD variations increased when using BodyFIX (0% for Butterfly Board compared with 25.8% for BodyFIX). The remaining BodyFIX re‐imaging incidence were due to either rectal or bladder‐filling issues so were not related to the stabilisation device used.

**Table 3:** Patients requiring re‐imaging on treatment or a planning CT re‐scan during treatment.

Treatment re‐imaging			
Patient cohort	Fractions when patient re‐imaged. Number (percentage)	Why the patient was re‐imaged. Number (percentage)	Fractions when patient re‐imaged. Number (percentage)
All patients	54/400 (13.5)	Pelvic tilt	15/54 (29.6)
		FSD variation	8/54 (14.8)
		Other	31/54 (57.4)
BodyFix patients	31/200 (15.5)	Pelvic tilt	6/31 (19.4)
		FSD variation	8/31 (25.8)
		Other	17/31 (54.8)
Butterfly Board patients	23/200 (11.5)	Pelvic tilt	9/23 (39.1)
		FSD variation	0/23 (0.0)
		Other	14/23 (60.9)

Planning CT re‐scan			
Patient cohort	Patients requiring planning CT re‐scan. Number (percentage)	Why the patient was re‐scanned. Number (percentage)	Patients requiring planning CT re‐scan. Number (percentage)
All patients	4/16 (25)	Pelvic tilt	1/16 (6.3)
		FSD variation	3/16 (18.8)
BodyFix patients	2/8 (25)	Pelvic tilt	0/8 (0)
		FSD variation	2/8 (25)
Butterfly Board patients	2/8 (25)	Pelvic tilt	1/8 (12.5)
		FSD variation	1/8 (12.5)

CT, computed tomography; FSD, focus to skin distance.

#### Planning image reviews and re‐scanning

Six of the 16 patients required at least one image review due to pelvic tilt during their treatment course. These were evenly distributed between the stabilisation methods. A total of four patients required a planning re‐scan during their course of treatment (Table 3). Only one patient required a re‐scan for pelvic tilt. This patient was stabilised using the Butterfly Board. Three patients required a re‐scan for FSD variations with two being stabilised using the BodyFIX system and one on the Butterfly Board.

### Survey responses

**Figure 4:**
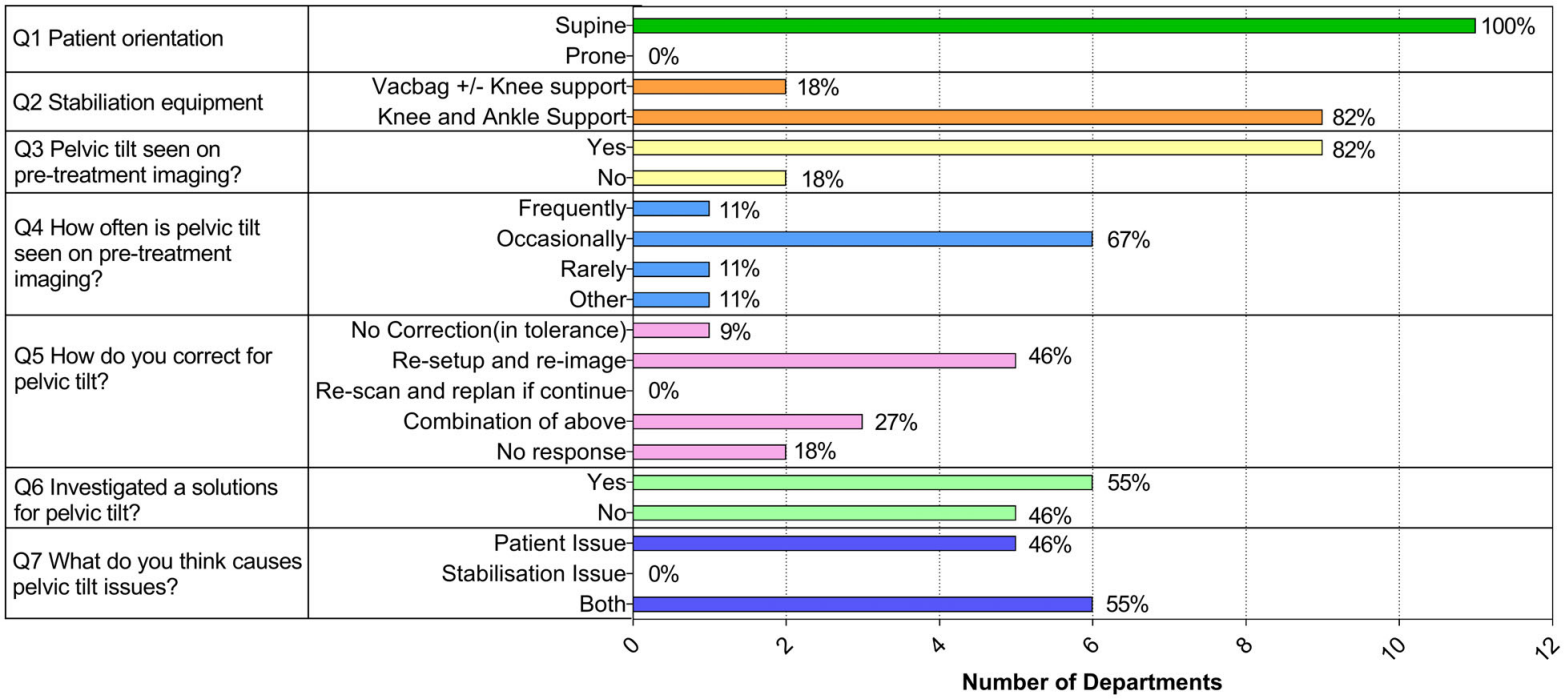
Survey responses from 11 departments about gynaecological radiotherapy stabilisation.

A total of 22 departments were sent the survey, and 11 departments responded (50%). Responses to the survey are presented in Figure 4. All departments treated patients in the supine position and only two other centres used some form of vacbag under the pelvis. Most departments treated with a form of knee block and ankle stocks under the legs with some centres treating with arms on chest and others with arms above head. 82% of departments (*n* = 9/11) reported seeing pelvic tilt on pre‐treatment imaging. Seven of the nine centres that see tilt on pre‐treatment imaging reported seeing tilt frequently or occasionally. In regard to correcting pelvic tilt, 8 of the 11 centres re‐setup and re‐image or do a combination of correction strategies. Fifty‐five per cent of the departments (*n* = 6/11) have tried to find a solution to pelvic tilt, and 55% of centres (*n* = 6/11) either thought tilt was caused by patient issues or by a combination of patient and stabilisation issues. Four centres in their free text answers indicated that they use six degrees of freedom couches to help correct pelvic tilt daily.

## Discussion

The use of the BodyFIX system reduced the pelvic tilt variance for gynaecological patients. This form of immobilisation also reduced the need for re‐imaging pre‐treatment. This could result in more accurate and efficient treatment of gynaecological external beam radiotherapy patients.

The results from the survey indicated that 82% of departments (*n* = 9/11) who responded to the survey reported seeing pelvic tilt on pre‐treatment imaging. Such results indicate that finding a strategy to tackle the problem is worth pursuing. This research indicates that using the BodyFIX system reduced re‐imaged fractions due to pelvic tilt by about half when compared to the Butterfly Board (39.1% for Butterfly Board compared to 19.4% for BodyFIX). This reduces the imaging dose delivered to the patient and the time the patient needs to be within the department. The decrease in intervention might also reduce treatment anxiety for patients.

A number of previous studies have investigated immobilisation devices for patients having pelvic radiotherapy. Comparison of patient‐specific and generic immobilisation devices for prostate radiotherapy indicated a slight increase in accuracy with the patient‐specific device, but concluded that image guidance may be the best way to correct inaccuracies.^10^ While assessing three‐dimensional patient setup error in uterine and cervical radiotherapy, Patni et al.^11^ noted that two different stabilisation devices were used during the time the images being reviewed were acquired. They noted that the mean shifts in the vertical, longitudinal and lateral directions were all slightly lower when using skin markings to set up rather than a thermoplastic shell.

There are a limited number of studies that have investigated pelvic tilt in pelvic patients. Kasabasic et al.^12^ investigated rotation of the sacrum during prone bellyboard pelvic radiotherapy for cervical, uterine and rectal cancer. They used daily portal film to measure the angle change during a course of radiotherapy and found that the setup error ranged from −14° to 11.5°. This is difficult to compare with the current study due to the variation in patient positioning between the two cohorts.

The timing of absolute differences ≥5° or 0.5 cm in pelvic tilt, pubic symphysis, sacral promontory and L5 spine was investigated at intervals of 1–5 fractions, 6–25 fractions and all fractions. This time frame was selected to assess whether differences in the first week of treatment could be used as an indicator of further differences. If this was the case, interventional re‐scans after five fractions could have been recommended for future patients with large differences. However, these differences vary throughout the treatment course. This indicates that fraction 1–5 are not a good indicator of what will occur during the treatment course. Therefore, daily correction is required for these patients.

An interesting finding was the requirement for more re‐scans for FSD variation when using BodyFIX for stabilisation. In reviewing these FSD changes, the majority occurred at the skin edge near the edge of the BodyFIX bag. This may be the result of trying to rotate the patient, with more skin being pulled anteriorly, or the skin folding anteriorly when the patient tries to enter the BodyFIX bag for each treatment. These issues could potentially be alleviated using slide sheets for rotation and having higher steps to allow the patient easier access for entering the BodyFIX bag.

The pubic symphysis, sacral promontory and L5 spine movement all contributed to the detected variance in pelvic tilt. Table 2 indicates that the more inferior bony anatomy, such as the pubic symphysis, had a slightly larger range of variance than the superior bony anatomy such as the L5 spine. However, due to the combined effect that this movement has on pelvic tilt, it is important to stabilise both areas of the pelvis.

Logistical issues also need to be considered when comparing these stabilisation devices. Trying to manoeuvre the patient during setup to correct pelvic tilt can be difficult for the radiation therapists. The use of BodyFIX could help reduce the amount of manual handling required, but the BodyFIX bags are large in size and require two radiation therapists to carry them between the storage area and the treatment couch. The BodyFIX bag can also be difficult for the patient to get in and out of due to the height of the edges of the moulded bag. Many require stairs to get on and off the treatment couch and this can be difficult for less mobile patients. The storage space required for the BodyFIX bags is also larger than that for the Butterfly Board which needs to be considered.

It is important to acknowledge that there are other tools available to assist with the correction of pelvic tilt apart from immobilisation. Yao et al.^13^ found the use of six degrees of freedom (6DoF) corrections with daily CBCT imaging improved positioning for gynaecological radiotherapy and allowed a reduction in planning target volume expansion, decreasing the normal tissue irradiated. The use of 6DoF cannot correct completely for excessive tilt and the combination of a rigorous immobilisation device and 6DoF correction might yield a more accurate setup. Surface‐guided radiation therapy (SGRT) might also assist with increased setup accuracy. A recent review of SGRT concluded that SGRT was a valuable tool in the initial setup of patients being irradiated in the pelvic area.^14^ SGRT might have been able to detect some of the FSD variation identified in this study.

There are a few limitations in this study. Firstly, given the limited numbers of gynaecologic patients treated in our centre each year, the small number of patients limits the robustness of statistical analysis. The allocation of the stabilisation device was not random because this was a retrospective analysis, but this could also impact the robustness of the statistical analysis. The patient age ranged vastly from 28 to 90 years with the median age being 10 years older in the BodyFIX cohort. Mobility could potentially impact setup variability; however, this was not assessed due to the retrospective nature of this study. We also did not evaluate organ motion or geographic miss during this investigation. The survey response rate was also low at 50%.

## Conclusions

The use of the BodyFIX system reduced the variance in pelvic tilt for gynaecological patients. This form of immobilisation also reduced the need for re‐imaging pre‐treatment by half, reducing imaging dose to the patients. Approximately 82% of departments who responded to the study survey reported seeing pelvic tilt on pre‐treatment imaging, indicating that it is worth finding a strategy to address this issue. The BodyFIX system could result in more accurate and efficient treatment of gynaecological external beam radiotherapy patients.

## Conflict of Interest

The authors declare no conflict of interest.

## Ethics

An ethics exemption was granted for this quality assurance/quality improvement project by the Northern Sydney Local Health District Human Research Ethics Committee.

## Supporting information


**Figure S1.** Measurements taken between the planning and treatment images.
**Table S1.** Survey questions.

## Data Availability

Research data are not shared.
