# Supplementary material for: Comparing immobilisation devices in gynaecological external beam radiotherapy: improving inter‐fraction reproducibility of pelvic tilt
Source: J Med Radiat Sci. 2024 Jun 19;71(4):529–39. doi: 10.1002/jmrs.804 (PMC11638372; doi:10.1002/jmrs.804)
Supplement: Supplementary file 1 — Figure S1. Measurements taken between the planning and treatment images. Table S1. Survey questions. [file JMRS-71-529-s001.docx]

**Supporting Information**

**Figure 1A:** Measurements taken between the planning and treatment images. Pelvic tilt was calculated by measuring the angle between the sacral promontory and the superior-anterior corner of the pubic symphysis on the reference image (a) and treatment image (b). Measurements of the pubic symphysis variations between the planning reference image and the daily acquired image were measured from the superior-anterior corner of the pubic symphysis in both the (c) AP and (d) SI directions. The variation in the sacral promontory was also measured in the (e) AP and (f) SI directions. If the superior-anterior corner of L5 was visible on both the planning reference image and the daily acquired image the (g) AP and (h) SI variation was also measured.

**
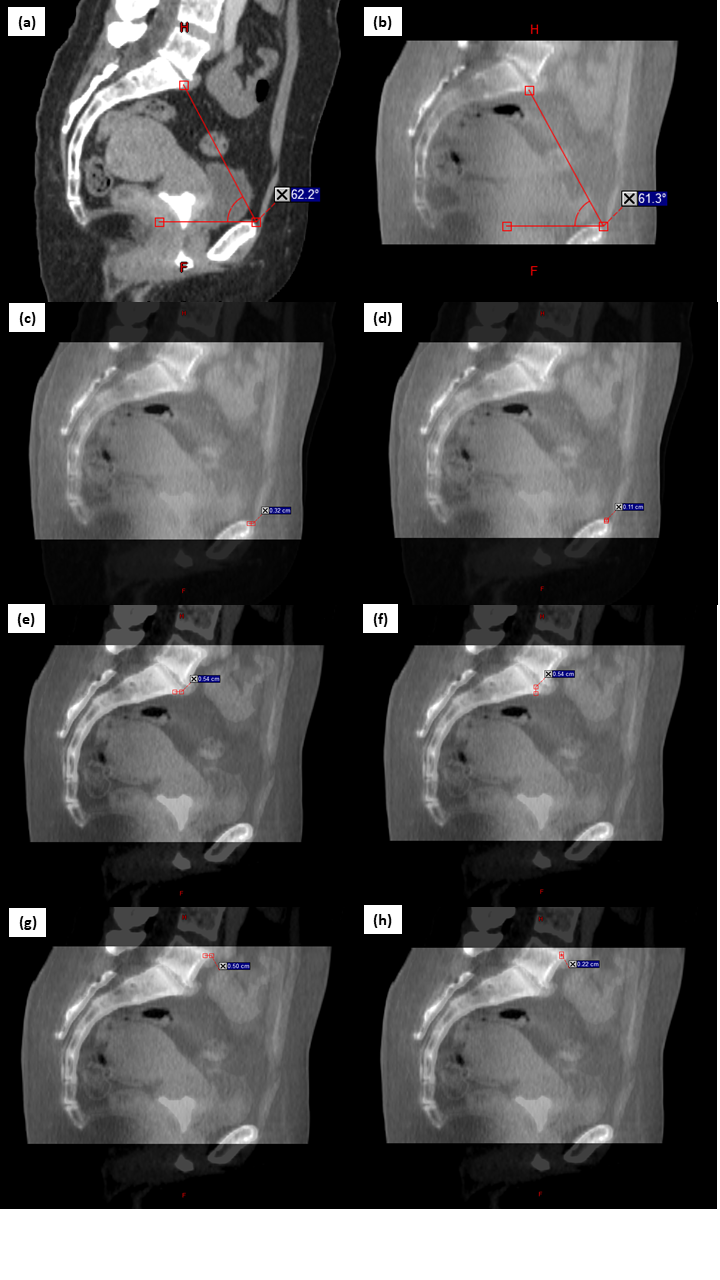
**

**Table1A:** Survey questions

| **Survey Questions** | |
| --- | --- |
| Question 1 | In your department, what patient orientation is routinely used for setting up patients for Gynae radiation therapy?   - 1. Supine   2. Prone |
| Question 2 | In your department, what stabilization equipment is used in setting up patients for Gynae radiation therapy? Please specify standard set-up protocol (i.e. Vac bag, knee fix etc). |
| Question 3 | At the time of pre-treatment imaging, have you experienced any pelvic tilt issues in this patient cohort?   1. Yes 2. No |
| Question 4 | How often is pelvic tilt observed in this patient cohort at the time of pre-treatment imaging?   1. Frequently 2. Occasionally 3. Rarely |
| Question 5 | How do you correct for pelvic tilt in this patient cohort?   1. No corrected if within RO accepted tolerance/department protocol 2. Re-setup and re-image 3. Re-scan and replan if tilt continues to be observed over consecutive fractions   If the above options do not cover what is done in your department, please explain how you deal with pelvic tilt. |
| Question 6 | Has your department investigated possible solutions for pelvic tilt?   1. Yes 2. No |
| Question 7 | Do you consider that the pelvic tilt is the result of a patient issue, stabilisation issue or both?   1. Patient Issue 2. Stabilisation Issue 3. Both   Please give details in the space below. |
